# A simple and efficient *Agrobacterium*‐mediated transient expression system to dissect molecular processes in *Brassica rapa* and *Brassica napus*

**DOI:** 10.1002/pld3.237

**Published:** 2020-07-06

**Authors:** Brian C. Mooney, Emmanuelle Graciet

**Affiliations:** ^1^ Department of Biology Maynooth University Maynooth Ireland; ^2^ Kathleen Lonsdale Institute for Human Health Research Maynooth University Maynooth Ireland

**Keywords:** agroinfiltration, *Brassica napus*, *Brassica rapa*, N‐degron pathway, transient expression

## Abstract

The family Brassicaceae is a source of important crop species, including *Brassica napus* (oilseed rape), *Brassica oleracea*, and *B. rapa*, that is used globally for oil production or as a food source (*e.g.,* pak choi or turnip). However, despite advances in recent years, including genome sequencing, a lack of established tools tailored to the study of *Brassica* crop species has impeded efforts to understand their molecular processes in greater detail. Here, we describe the use of a simple *Agrobacterium*‐mediated transient expression system adapted to *B. rapa* and *B. napus* that could facilitate study of molecular and biochemical events in these species. We also demonstrate the use of this method to characterize the N‐degron pathway of protein degradation in *B. rapa*. The N‐degron pathway is a subset of the ubiquitin‐proteasome system and represents a mechanism through which proteins may be targeted for degradation based on the identity of their N‐terminal amino acid residue. Interestingly, N‐degron‐mediated processes in plants have been implicated in the regulation of traits with potential agronomic importance, including the responses to pathogens and to abiotic stresses such as flooding tolerance. The stability of transiently expressed N‐degron reporter proteins in *B. rapa* indicates that its N‐degron pathway is highly conserved with that of *Arabidopsis thaliana*. These findings highlight the utility of *Agrobacterium*‐mediated transient expression in *B. rapa* and *B. napus* and establish a framework to investigate the N‐degron pathway and its roles in regulating agronomical traits in these species.

**Significance statement:**

We describe an *Agrobacterium‐*mediated transient expression system applicable to *Brassica* crops and demonstrate its utility by identifying the destabilizing residues of the N‐degron pathway in *B. rapa*. As the N‐degron pathway functions as an integrator of environmental signals, this study could facilitate efforts to improve the robustness of *Brassica* crops.

## 1 INTRODUCTION

The *Brassica* genus is a source of important crops, including *B. napus* (oilseed rape), *B. oleracea* (broccoli and cauliflower), and *B. rapa*. *B. napus* is an allotetraploid and originated from a hybridization between *B. oleracea* and *B. rapa*, two diploid species (Allender & King, 2010; Lu et al., 2019). *B. napus* and *B. rapa* are grown globally mainly for vegetable oil derived from their seeds. Both crops are also widely used as fodder for livestock, while some *B. rapa* cultivars are grown to produce food for human consumption including pak choi and turnip. Despite their similarities, certain characteristics of each species determine their suitability for cultivation in particular regions. Over the last decade, research with *Brassica* crop species has been facilitated by the sequencing of their genomes, including different accessions of a single species (Chalhoub et al., 2014; Golicz et al., 2016; Lu et al., 2019; Wang et al., 2011; Yang et al., 2016; Zhang, Cai, et al., 2018). Another asset for the establishment of *Brassica* crop species as model systems is the development of Targeting Induced Local Lesions IN Genomes (TILLING) collections (Gilchrist et al., 2013; Himelblau et al., 2009; Stephenson et al., 2010), which can be used for either reverse or forward genetics approaches (Chen, Hao, Parry, Phillips, & Hu, 2014) using non‐transgenic plants. Various protocols for the stable transformation of *Brassica* crops have also been developed. However, many of these protocols involve *Agrobacterium* co‐cultivation followed by plant regeneration from callus (De Block, De Brouwer, & Tenning, 1989; Sanimah, Sundakar, Linthorst, & Verpoorte, 2010; Sparrow, Dale, & Irwin, 2006), which makes them difficult to implement because of their labor intensiveness and the need for tissue culture. A simple floral dip method has also been established for *B. napus* (Verma, Chinnusamy, & Bansai, 2008). While this approach facilitates the transformation procedure and the isolation of transformants, the generation time of *Brassica* crop species remains a bottleneck to dissect mechanisms swiftly.

In contrast to stable lines, transient expression methods enable rapid experiments, thus facilitating and accelerating the elucidation of mechanisms and pathways. Although transient expression approaches are often applied using heterologous systems (*e.g.,* using *Nicotiana benthamiana* as a model), the ability to examine molecular and biochemical mechanisms within a homologous system has significant advantages. For example, the function of a *B. rapa* protein in *N. benthamiana* may be limited if *B. rapa*‐specific co‐factors or interacting proteins are required for the activity of the protein of interest. Transient expression may be achieved through a range of methods, including the use of mesophyll protoplasts, projectile bombardment, or *Agrobacterium tumefaciens* to introduce a transfer‐DNA (T‐DNA) of interest into leaf or root tissue. *Agrobacterium*‐mediated approaches offer several advantages in that they are easy to implement, do not require specialized laboratory equipment, and do not involve the isolation of protoplasts from leaf tissue, which can be challenging. Despite this, the development of transient expression approaches in *Brassica* crop species has so far been hindered by low transformation efficiency or the need for biolistic equipment, although limited examples of agroinfiltration methods have emerged in recent years (Das, Naiya, Marik, Mukherjee, & Seal, 2019; Zhong et al., 2016).

Here, we present a transient expression protocol based on the infiltration of *Agrobacterium* into the leaves of *B. rapa*. We also show that this protocol is easily applicable to *B. napus*. To demonstrate its utility for the dissection of biological pathways, we employed transient expression in *B. rapa* to uncover the sets of N‐terminal residues that can serve as degradation signals (degrons) for N‐degron pathway‐mediated degradation. The N‐degron pathway is a conserved ubiquitin‐dependent protein degradation pathway in eukaryotes. In yeast and mammals, different branches of the pathway have been characterized (Varshavsky, 2019), but in plants, the so‐called PRT6/N‐degron and PRT1/N‐degron branches have been the main focus of research (reviewed in Dissmeyer, 2019; Dissmeyer, Rivas, & Graciet, 2018; Gibbs, Bacardit, Bachmair, & Holdsworth, 2014; Gibbs, Bailey, Tedds, & Holdsworth, 2016). Substrate proteins of the PRT6 and PRT1/N‐degron pathways are targeted for degradation based on the identity of their N‐terminal residue. More specifically, exposure of the so‐called destabilizing N‐terminal residues (*e.g.,* following protease cleavage) can result in their recognition by E3 ubiquitin ligases of the N‐degron pathway (termed N‐recognins), that catalyze the conjugation of a ubiquitin chain to the substrate followed by its degradation by the 26S proteasome. The PRT6 and PRT1/N‐degron pathways play an important role in the regulation of plant responses to environmental and developmental cues such as light, oxygen, and photoperiod (Abbas et al., 2015; Dong et al., 2017; Gibbs et al., 2011, 2018; Goslin et al., 2019; Graciet et al., 2009; Holman et al., 2009; Labandera et al., 2020; Licausi et al., 2011; Till et al., 2019; Valeri et al., 2020; Weits et al., 2019; Zhang, Gannon, Hassall, et al., 2018). Interestingly, recent studies have highlighted the relevance of this pathway for the regulation of traits of agronomic importance. For example, *Arabidopsis* and barley (*Hordeum vulgare*) plants in which the function of the N‐recognin PROTEOLYSIS6 (PRT6) is impaired display higher tolerance to waterlogging due to the constitutive accumulation of group VII Ethylene Response Factors (ERF‐VII), transcription factors that act as master regulators of the hypoxia response program (Gibbs et al., 2011; Mendiondo et al., 2016). *Arabidopsis* plants mutant for *PRT6* are also more tolerant to a range of abiotic stresses, including drought, high salt, and high temperature (Holdsworth, Vicente, Sharma, Abbas, & Zubrycka, 2020; Vicente et al., 2017). In addition, the N‐degron pathway has been implicated in the regulation of plant defense against pathogens (Gravot et al., 2016; de Marchi et al., 2016; Vicente et al., 2019) and of hormone signaling pathways (such as jasmonic acid and abscisic acid) that mediate plant responses to both biotic and abiotic stresses (Holman et al., 2009; de Marchi et al., 2016; Zhang, Gannon, Jones, et al., 2018). The N‐degron pathway thus appears to function as an integrator of environmental signals (Miricescu, Goslin, & Graciet, 2018), and greater understanding of its substrate specificity could assist efforts to develop improved crop varieties. Here, we identified N‐degron pathway components in *B. rapa* and uncovered the sets of destabilizing residues using *Agrobacterium‐*mediated transient expression. In sum, our results not only validate the use of the agroinfiltration method for transient expression in *Brassica* crops but also provide a framework for the study of the N‐degron pathway in these crops.

## 2 MATERIALS AND METHODS

### 2.1 Plant materials and growth conditions

All *Brassica rapa* subsp. *trilocularis* used in this study were of the R‐o‐18 genotype (Yellow Sarson). *Brassica napus* were of the “Westar” cultivar. Seeds for agroinfiltration experiments were sown on a soil:vermiculite:perlite (5:3:2; v:v:v) mixture and stratified at 4°C for 3 days. These seeds were germinated and grown in continuous light conditions (24‐hr light) at 20°C for 7 days before being transferred to short‐day conditions (10‐hr light/14‐hr dark) at 22°C for a further 3–4 weeks. For the co‐cultivation experiments, seeds were planted on 0.5x MS agar medium and stratified at 4°C for 3 days. Seedlings were grown in continuous light conditions (24 hr light) at 20°C for 3 days before starting the co‐cultivation.

### 2.2 N‐degron pathway reporter constructs

All N‐degron pathway reporter constructs used were previously published (Graciet, Mesiti, & Wellmer, 2010; Worley, Ling, & Callis, 1998; Yoshida, Ito, Callis, Nishida, & Watanabe, 2002). pCAMBIA2201 was used to assess the expression of GUS containing the *cat1* intron.

### 2.3 Co‐cultivation assays

*B. rapa* seeds were planted on 15‐ml 0.5x MS agar medium in 50‐ml centrifuge tubes and seedlings were grown in constant light at 20°C for 3 days. A previously published co‐cultivation protocol established for use with *Arabidopsis thaliana*, as well as tobacco, tomato, and rice (Li & Nebenfuhr, 2010), was modified for *B. rapa* as indicated below. *A. tumefaciens* (C58 pGV2260 [McBride & Summerfelt, 1990]) transformed with the N‐degron pathway reporter construct pML‐BART UBQ3_pro_:Ub‐Gly‐LUC 35S_pro_:GUS (pEG378; Graciet et al., 2010) was streaked from glycerol stock on LB medium supplemented with 50 mg/L rifampicin, 100 mg/L ampicillin, and 100 mg/L spectinomycin and grown for 2–3 days at 28°C before being suspended in washing solution (10 mM MgCl_2_, 100 µM acetosyringone). Seven individual 3‐day‐old *B. rapa* seedlings were transferred to a 50‐ml centrifuge tube containing 30‐ml co‐cultivation medium (1.13g/L MS medium, 1% sucrose (w/v), 100 µM acetosyringone, 0.001% Silwet (v/v), pH6.0), and *Agrobacteria* transformed with the indicated plasmids were added to a final OD_600_ of 0.5. Samples were then vacuum infiltrated at 80 kPa for 10–20 min. Tubes were wrapped in aluminum foil and incubated at 20°C for 30–48 hr of co‐cultivation. Seedlings were washed with autoclaved ddH_2_O three times and placed on 0.5x MS agar plates for 24 hr before GUS staining procedure.

### 2.4 Agroinfiltration of *B. rapa* and *B. napus*

*A. tumefaciens* C58 pGV2260 (McBride & Summerfelt, 1990) transformed with the indicated N‐degron pathway reporters or a pML‐BART empty vector were grown for 3–4 days at 28°C on LB agar supplemented with 50 mg/L rifampicin, 100 mg/L ampicillin, and 100 mg/L spectinomycin. After 3–4 days growth, bacteria were suspended from plates in 2‐ml infiltration medium (10 mM MES pH5.5, 10 mM MgCl_2_, 150 µM acetosyringone) and diluted to OD_600_ of 0.75. Four‐ to five‐week‐old *B. rapa* or *B. napus* were covered with plastic lids overnight prior to infiltration. A ~2 cm diameter area was marked on the abaxial side of the first and second true leaves. Using a blunt 1‐ml syringe, the bacterial suspension was infiltrated into the marked areas. Excess liquid was removed with tissue paper and plants were returned to the growth room. Unless otherwise stated, tissue was harvested 3 days post‐agroinfiltration for GUS staining or protein extraction.

### 2.5 GUS staining

GUS staining was performed as described in Jefferson, Kavanagh, and Bevan (1987) using 2 mM X‐Gluc (5‐bromo‐4‐chloro‐3‐indolyl‐beta‐D‐glucuronic acid, cyclohexylammonium salt; Thermo Scientific) as a substrate.

### 2.6 Enzymatic assays to analyze GUS and LUC activity

For LUC and GUS assays, four agroinfiltrated leaf discs (diameter: 1 cm) per construct were pooled and proteins were extracted in 450 µL 1x CCLR buffer (Promega) supplemented with 1 mM phenylmethylsulfonyl fluoride (PMSF; Sigma‐Aldrich) and 1% (v/v) plant protease inhibitor cocktail (Sigma‐Aldrich). LUC activity was measured as described in Luehrsen, de Wet, and Walbot (1992) and Graciet et al. (2010). Protein extract (1.5 or 2 µl) was added to 100 µl LAR buffer (20 mM tricine pH7.8, 1.07 mM (MgCO_3_)_4_Mg(OH)_2_.5H_2_O, 2.67 mM MgSO_4_, 0.1 mM ethylenediaminetetraacetic acid (EDTA), 33.3 mM dithiothreitol (DTT), 270 µM coenzyme A, 470 µM luciferin, 530 µM ATP). Luminescence was measured using a POLARstar Omega microplate reader (BMG LABTECH) for 10 s.

GUS activity was quantified using 4‐methylumbelliferyl‐β‐D‐glucuronide (MUG) as described in Weigel and Glazebrook (2002) and Graciet et al. (2010). Protein extract (20 µL) in CCLR buffer was added to 150 µL of the GUS reaction mixture (50 mM sodium phosphate buffer pH 7.0, 10 mM, EDTA, 0.1% (v/v) sodium dodecyl sulfate (SDS), 0.1% (v/v)Triton X100). The fluorescent product 4‐methylumbelliferone (4‐MU) was measured 10, 20, 30, and 40 min after the initiation of the reaction using the POLARstar Omega instrument (BMG LABTECH). These values were calibrated against a standard curve prepared with known concentrations of 4‐MU ranging from 12.5 to 400 µM.

Differences in the accumulation of the different N‐degron reporter constructs relative to that of Met‐LUC were tested using an unpaired Student's *t* test and Welch correction because of unequal sample size and variation, with a *P* < .05.

### 2.7 Cloning of pET22b His_6_‐Ub‐Met‐LUC

The Ub‐Met‐LUC sequence was excised from p4202 (Worley et al., 1998) using *Nco*I/*BamH*I. The fragment was then ligated into pEG429 that had been digested with *Nco*I/*BamH*I to yield pEG432. The plasmid pEG429 is a derivative of pET22b, in which the pelB sequence was replaced by the sequence of a polyhistidine (6xHis) tag. This was achieved by cutting pET22b with *Nde*I/*Nco*I and annealing the oligonucleotides His6_up and His6_lo to create an insert coding for a polyhistidine tag before ligating into pET22b.

### 2.8 Expression of His_6_‐Ub‐Met‐LUC in *E. coli*

pET22b His_6_‐Ub‐Met‐LUC (pEG432) was transformed into *E. coli* BL21(DE3) pLysS RARE. Fresh colonies were used to inoculate 250 ml LB supplemented with 100 mg/L ampicillin and 35 mg/L chloramphenicol. Cells were grown at 37°C to an OD_600_ of 0.5, then cooled on ice for 30 min. Expression of His_6_‐Ub‐Met‐LUC was induced by adding 0.5 mM isopropyl β‐D‐1‐thiogalactopyranoside (IPTG). The culture was then grown for 5 hr at 30°C. Cells were harvested by centrifugation and frozen at −80°C. Proteins were extracted by resuspending part of the pellet into 2x SDS loading buffer (Laemmli buffer).

### 2.9 Detection of LUC and GUS reporter proteins by immunoblot

Agroinfiltrated leaf tissue was harvested and frozen immediately in liquid nitrogen. Proteins from *B. rapa* were extracted using 6x SDS loading buffer (Laemmli buffer) with 80 µl of buffer used per 1.5 cm diameter leaf disc. Proteins were separated on a 10% SDS‐PAGE gel before being transferred to a PVDF membrane. LUC was detected using a goat antibody against firefly Luciferase (AB3256, Merck) diluted 1:2,000 in PBS‐T (1x PBS with 0.05% Tween 20 [v/v]) containing 5% milk (w/v). After stripping, the same membranes were probed with the rabbit anti‐GUS antibody (A5790; Invitrogen) at a 1:1,000 dilution in PBS‐T with 5% milk. To observe LUC expression in samples with approximately equal GUS levels, the protein quantity loaded was adjusted based on previous immunoblots with replicate samples.

### 2.10 Alignments and gene expression analysis

*B. rapa* homologs of *Arabidopsis* N‐degron pathway components were identified using BLASTp at Ensembl with the protein sequence of *Arabidopsis* enzymes as a query (At*PCO1* (At5g15120); At*NTAQ1* (AT2G41760); At*NTAN1* (At2g44420); At*ATE1* (At5g05700); At*ATE2* (At3g11240); At*PRT6* (At5g02310); At*PRT1* (AT3G24800)). Protein sequences were aligned using ClustalX. Panels in Figure 5 were generated using BoxShade 3.2 using ClustalX output files (https://embnet.vital‐it.ch/software/BOX_form.html).

The tissue‐specific gene expression of the N‐degron pathway‐related genes in *B. rapa* was obtained from a publicly available dataset (GEO43245) (Tong et al., 2013). The heatmap was generated using HeatMapper tool (available at: https://bar.utoronto.ca/).

## 3 RESULTS

### 3.1 Adapting an *Agrobacterium* co‐cultivation method for transient expression in *B. rapa*

**FIGURE 1:**
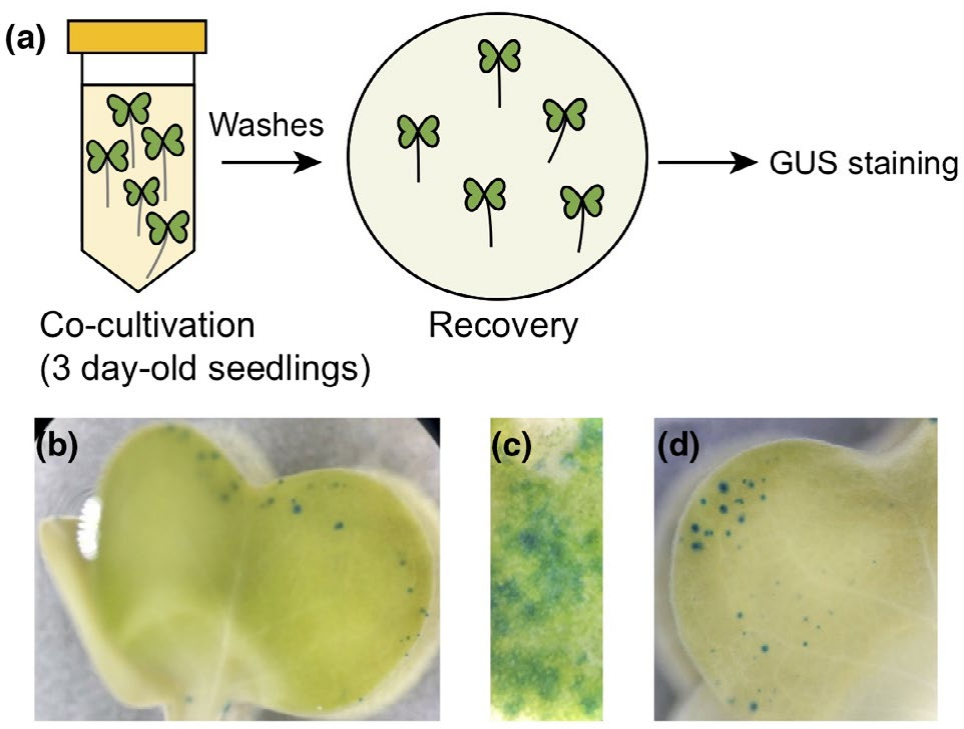
An *Agrobacterium*‐mediated co‐cultivation method applied to *Brassica rapa*. (a) Overview of the co‐cultivation method. Three‐day‐old *B. rapa* R‐o‐18 seedlings were co‐cultivated with an *Agrobacterium* suspension at an OD_600_ of 0.5 in co‐cultivation medium supplemented with 0.001% Silwet (v/v). Seedlings were collected 24 hr after the beginning of the recovery period. (b) Expression of a GUS reporter gene from the 35S promoter in cotyledons after 30 hr of co‐cultivation and 24‐hr recovery. (c) Close‐up on a region with high GUS expression using the same experimental procedure as in (b). Picture taken from a different seedling as in (b). (d) Expression of a GUS reporter gene from the 35S promoter in cotyledons after 40 hr of co‐cultivation and 24‐hr recovery

Co‐cultivation of *A. tumefaciens* transformed with a plasmid coding for a T‐DNA of interest has been shown to allow transient expression of transgenes in the model plant *Arabidopsis thaliana* (also a Brassicaceae), although with variable efficiency depending on the genotype used (Li, Park, von Arnim, & Nebenfuhr, 2009; Wu et al., 2014). In brief, these transient expression methods rely on the co‐cultivation of young seedlings with *Agrobacterium* for several days. After washing the seedlings to remove *Agrobacterium* cells, seedlings are transferred to MS agar medium (*i.e.,* recovery period), during which the transgene(s) may be transiently expressed (Figure 1a). The use of young seedlings in co‐cultivation methods has the potential to speed up transient expression experiments while also allowing analysis in either roots or aerial organs.

To test if a co‐cultivation method could lead to successful transient expression in *Brassica*, we used 3‐day‐old *B. rapa* seedlings as a model to express a GUS reporter gene under the control of an enhanced version of the constitutive Cauliflower Mosaic Virus 35S promoter. We tested the effects of varying concentrations of Silwet in the co‐cultivation medium (0.001%–0.005% [v/v]), different co‐cultivation (30–48 hr) times, as well as the use of vacuum infiltration or not. Recovery time was kept constant at 24 hr. Overall, the use of low Silwet concentrations (0.001%) improved seedling survival during co‐cultivation, with higher doses resulting in limp seedlings that senesced during the 24‐hr recovery period (data not shown). Similarly, shorter co‐cultivation times were beneficial for seedling health and recovery. For example, after a 30‐hr co‐cultivation period, seedlings remained green while after 40 or 48 hr of co‐cultivation, seedlings appeared wilted and pale (data not shown). Irrespective of the co‐cultivation duration, only small patches of cells expressing the GUS reporter could be observed in the cotyledons of co‐cultivated seedlings (Figure 1b–d). To establish a more efficient protocol that would yield expression in more cells, we next sought to develop an agroinfiltration‐based assay of *Brassica* leaves.

### 3.2 Establishing an agroinfiltration protocol for transient expression in *Brassicas*

**FIGURE 2:**
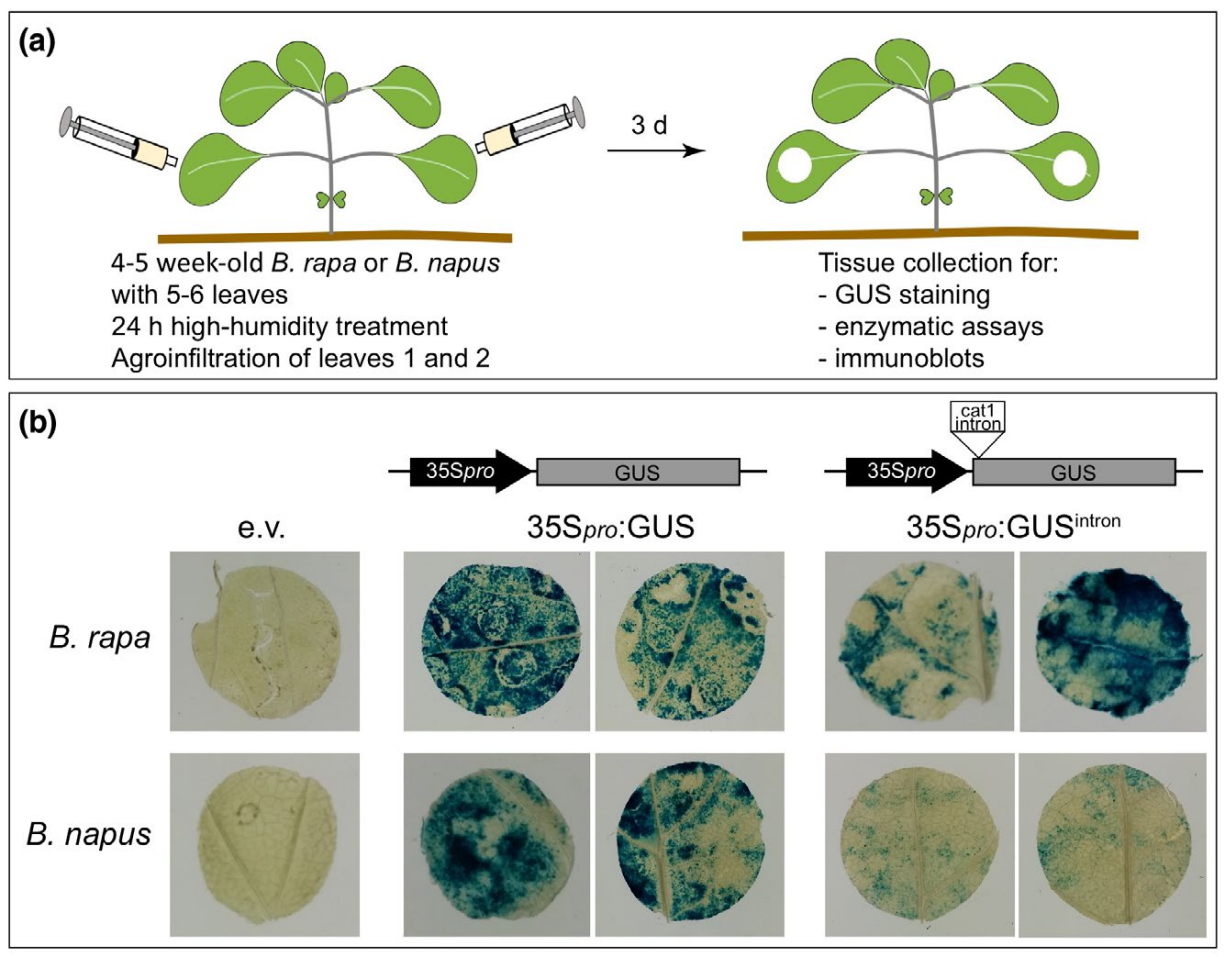
Transient expression *via* agroinfiltration in *Brassica rapa* and *Brassica napus*. (a) Overview of the agroinfiltration method used in *B. rapa* and *B. napus*. A suspension of *Agrobacterium* at an OD_600_ of 0.75 was infiltrated into leaves 1 and 2 of 4‐ to 5‐week‐old plants (see Materials and Methods for growth conditions). Leaf tissue was harvested 3 d after agroinfiltration for analyses. (b) Representative GUS stains of *B. rapa* and *B. napus* leaf discs agroinfiltrated with the indicated constructs: empty vector (e.v.), pEG356 or pEG368 (coding for 35S_pro_:GUS) or pCAMBIA2201 (coding for 35S:GUS^intron^). Leaves 1 and 2 of 4‐ to 5‐week‐old plants grown in short‐day conditions. Leaf tissue was harvested 3 d after agroinfiltration for analyses (leaf discs were either 1 or 1.5 cm in diameter). The same experiments were performed at least 3 times independently with similar results qualitatively

We conducted agroinfiltration experiments using *B. rapa* plants grown in walk‐in growth chambers (see Materials and Methods for growth conditions) using leaves number 1 and 2 of 4‐ to 5‐week‐old plants that had formed 5–6 leaves on average (Figure 2a and Figure S1). Our results showed that the transient expression of the GUS reporter worked well under these conditions (Figure 2b). We also validated the use of our protocol in *B. napus* (variety: Westar) using leaves 1 and 2 of 4‐ to 5‐week‐old plants (Figure S1) grown under similar conditions as *B. rapa* (Figure 2b), thus extending the potential applications for the transient expression system. Additionally, we tested the expression of a GUS reporter that carried the *cat1* intron inserted after the first 15 bases of the GUS coding sequence (35S_pro_:GUS^intron^) to confirm that GUS signals originated from transient expression *in planta* as opposed to potential leaky expression by the *Agrobacterium* strains used to infiltrate the plants. Expression of 35S_pro_:GUS^intron^ was detected in both *B. rapa* and *B. napus* (Figure 2b). In both species, expression of 35S_pro_:GUS^intron^ was however weaker than that of the 35S_pro_:GUS construct, likely due to the presence of an enhanced 35S promoter in the 35S_pro_: GUS construct (Kay, Chan, Daly, & McPherson, 1987; Norris, Meyer, & Callis, 1993; Worley et al., 1998), which can lead to 10‐fold higher levels of expression than a typical 35S promoter such as the one present upstream of GUS^intron^ (Figure 2b).

**FIGURE 3:**
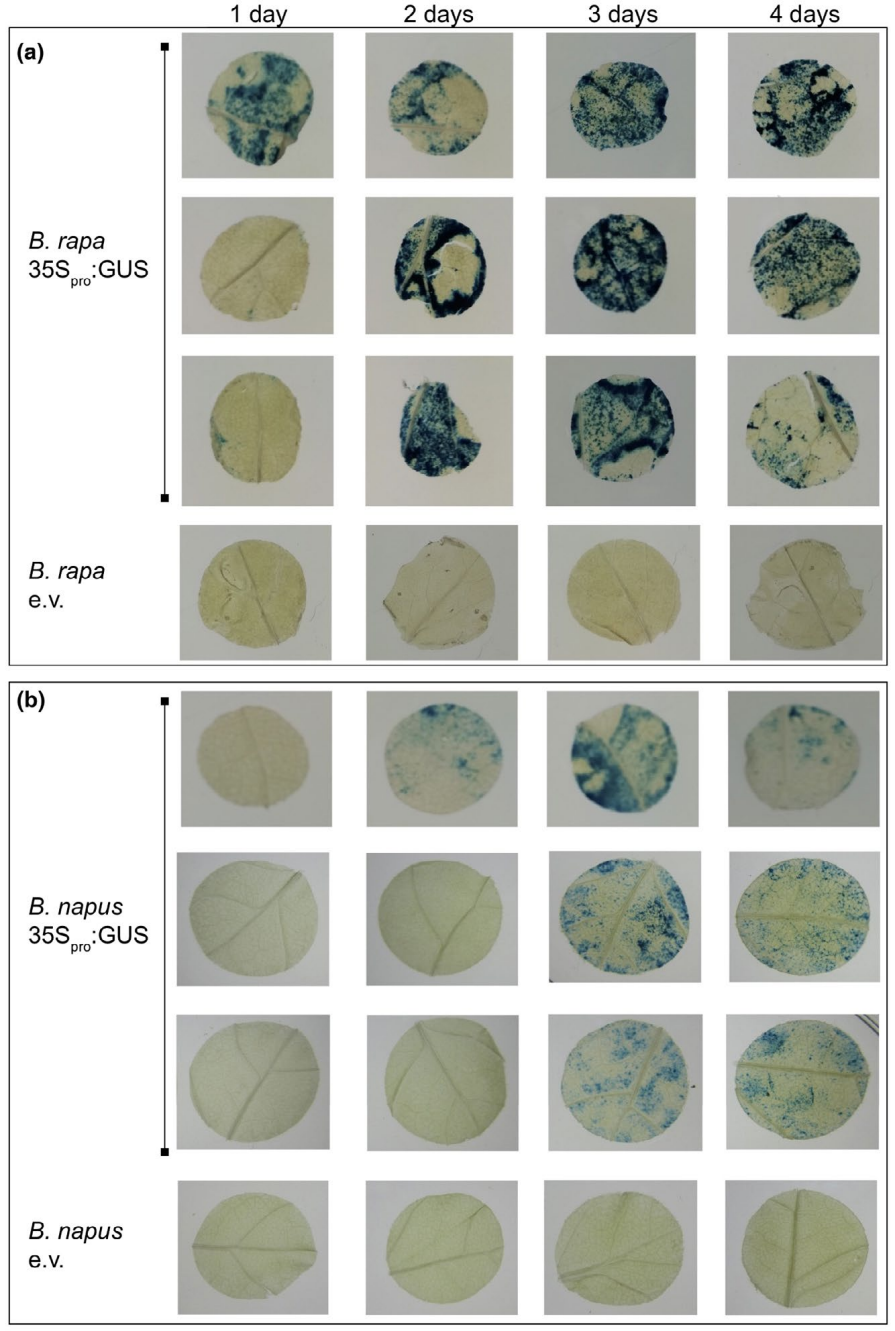
Time course experiment for expression of a GUS reporter. (a) Representative GUS stains of *Brassica rapa* leaf discs agroinfiltrated with *Agrobacterium* transformed with the indicated constructs: empty vector (e.v.) or a plasmid coding for 35S_pro_:GUS (pEG356 or pEG368). (b) Representative GUS stains of *Brassica napus* leaf discs agroinfiltrated with *Agrobacterium* transformed with a plasmid coding for 35S_pro_:GUS (pEG356 or pEG368). For (a) and (b): a suspension of *Agrobacterium* at an OD_600_ of 0.75 was infiltrated into leaves 1 and 2 of 4‐ to 5‐week‐old plants (see Materials and Methods for growth conditions). Leaf discs (either 1 or 1.5 cm in diameter) of infiltrated tissue were collected 1, 2, 3, or 4 days after agroinfiltration. The same experiments were performed at least three times independently with similar results qualitatively

Next, we conducted a time course experiment to determine the onset and duration of GUS expression after agroinfiltration. Our results show that in *B. rapa*, expression may be detected as soon as 24‐hr post‐agroinfiltration, although in most cases, it took up to 2 days to detect GUS activity (Figure 3a). GUS activity was sustained for at least 4 days after agroinfiltration. Notably, similar results were observed with *B. napus* (Figure 3b), thus further validating the transient expression system for use in this crop.

### 3.3 Establishing the sets of stabilizing and destabilizing residues of the N‐degron pathway in *Brassicas*

**FIGURE 4:**
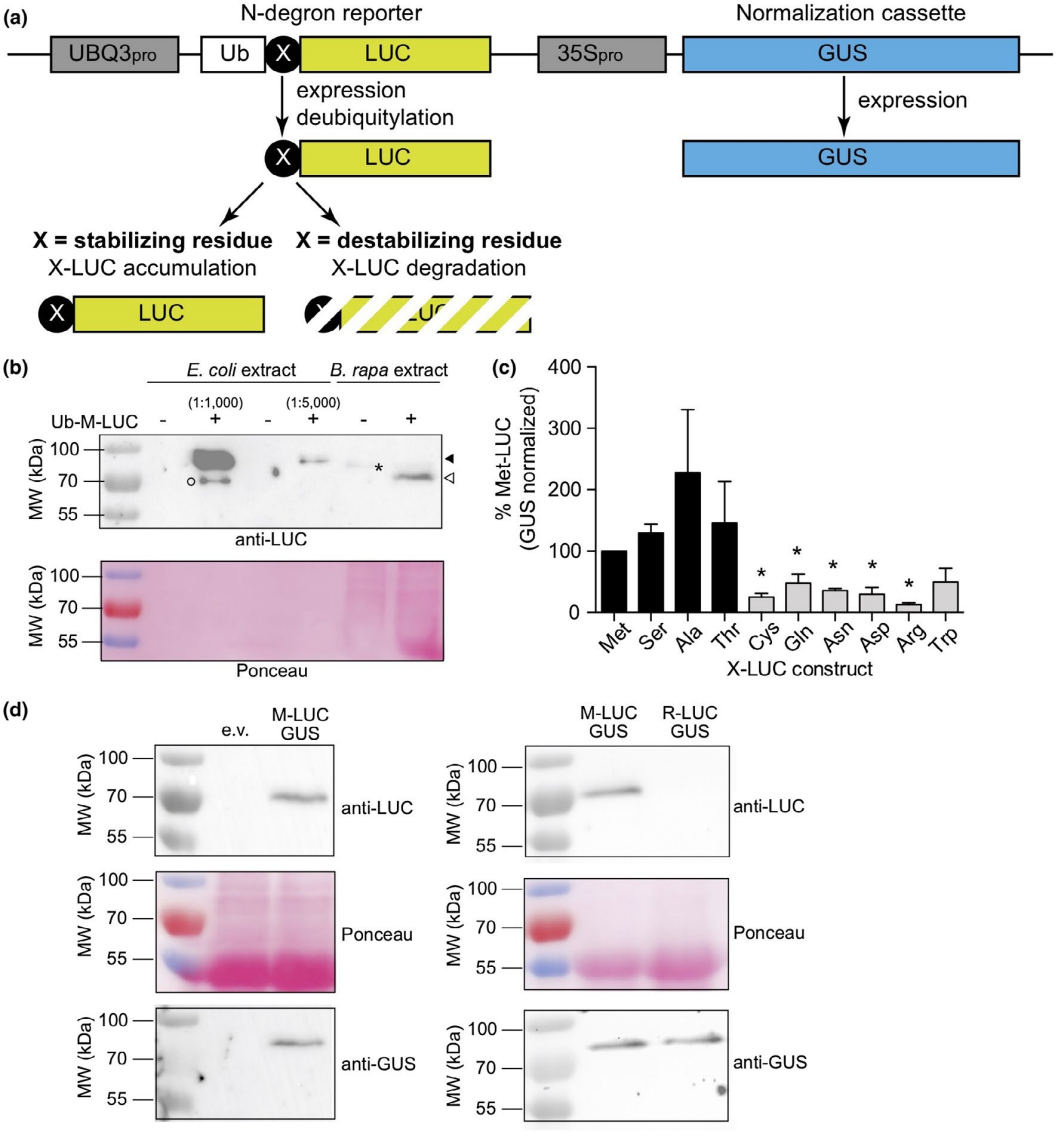
N‐degron pathway reporters and destabilizing residues in *Brassica rapa*. (a) Components of N‐degron pathway reporter constructs (Graciet et al., 2010; Worley et al., 1998), including the Ub‐X‐LUC fusion (“X” denoting any amino acid residue) and the normalization cassette (a GUS reporter under the control of an enhanced 35S promoter (35S_pro_)). (b) In vivo deubiquitylation of Ub‐Met‐LUC in *B. rapa*. A polyhistidine‐tagged His_6_‐Ub‐Met‐LUC was expressed in *E. coli* as a control for a non‐deubiquitylated N‐degron reporter. Different dilutions (1:1,000 and 1:5,000) of the crude *E. coli* protein extracts were loaded to ensure comparable signal intensities with *B. rapa* samples. Protein extracts from *B. rapa* tissue infiltrated with *Agrobacterium* transformed with empty vector (background control) or pEG356 (UBQ3_pro_:Ub‐Met‐LUC and 35S_pro_:GUS) were also loaded. Expected molecular weights: His_6_‐Ub‐Met‐LUC: 72 kDa (black arrowhead); Ub‐Met‐LUC: 71 kDa; Met‐LUC: 62 kDa (open arrowhead). Asterisk: cross‐reacting protein; open circle: potential degradation product of His_6_‐Ub‐Met‐LUC in *E. coli*. Original blots are shown in Figure S2. Results are representative of at least three independent replicates. (c) Stability of X‐LUC N‐degron reporters in *B. rapa*. LUC activities were normalized against the corresponding GUS activities (non‐normalized results shown in Figure S3) and expressed relative to that of Met‐LUC for each of the replicates. Error bars represent standard errors of four independent replicates, except for Asn (two independent replicates). Black and gray bars correspond to stabilizing and destabilizing N‐terminal residues, respectively. Asterisks denote statistically significant differences (*P* < .05) to the levels of Met‐LUC (determined using Student's *t* test with a Welch correction). (d) Immunoblot analysis of LUC and GUS protein levels for N‐terminal Met and Arg. Left: approximately equal protein amounts were loaded to determine potential cross‐reacting proteins in *B. rapa* infiltrated with *Agrobacterium* transformed with an empty vector (e.v.) or pEG356 (Ub‐Met‐LUC). Right: comparison of Met‐LUC and Arg‐LUC signal intensities for equal GUS levels after infiltration with *Agrobacterium* transformed with pEG356 (Ub‐Met‐LUC) or pEG368 (Ub‐Arg‐LUC). Results are representative of at least three independent replicates

To validate the use of the agroinfiltration protocol, we transiently expressed N‐degron reporter constructs to determine the sets of N‐terminal destabilizing residues that serve as degradation signals for the PRT6 and PRT1/N‐degron pathways in *B. rapa*. These previously developed and characterized N‐degron reporter constructs (Graciet et al., 2010; Worley et al., 1998) rely on the expression of a fusion protein composed of ubiquitin (Ub), a variable residue X and the reporter protein luciferase (Ub‐X‐LUC) under the control of the *Arabidopsis* UBQ3 promoter (Figure 4a). Upon expression, the Ub‐X‐LUC fusion protein is cleaved after the last residue of Ub by deubiquitylating enzymes, thus releasing a LUC reporter protein that starts with a defined N‐terminal residue “X.” The effect of the N‐terminal residue “X” on the stability of the LUC reporter can be determined either by quantitatively measuring LUC activity or by immunoblot, with the exposure of an N‐terminal destabilizing residue X resulting in low levels of LUC (Graciet et al., 2010; Worley et al., 1998; Yoshida et al., 2002). Each T‐DNA also codes for a normalization cassette (i.e., a GUS reporter gene under the control of an enhanced 35S promoter) to normalize the LUC activities obtained for different reporter constructs and also across different plants and biological replicates (Worley et al., 1998).

**FIGURE 5:**
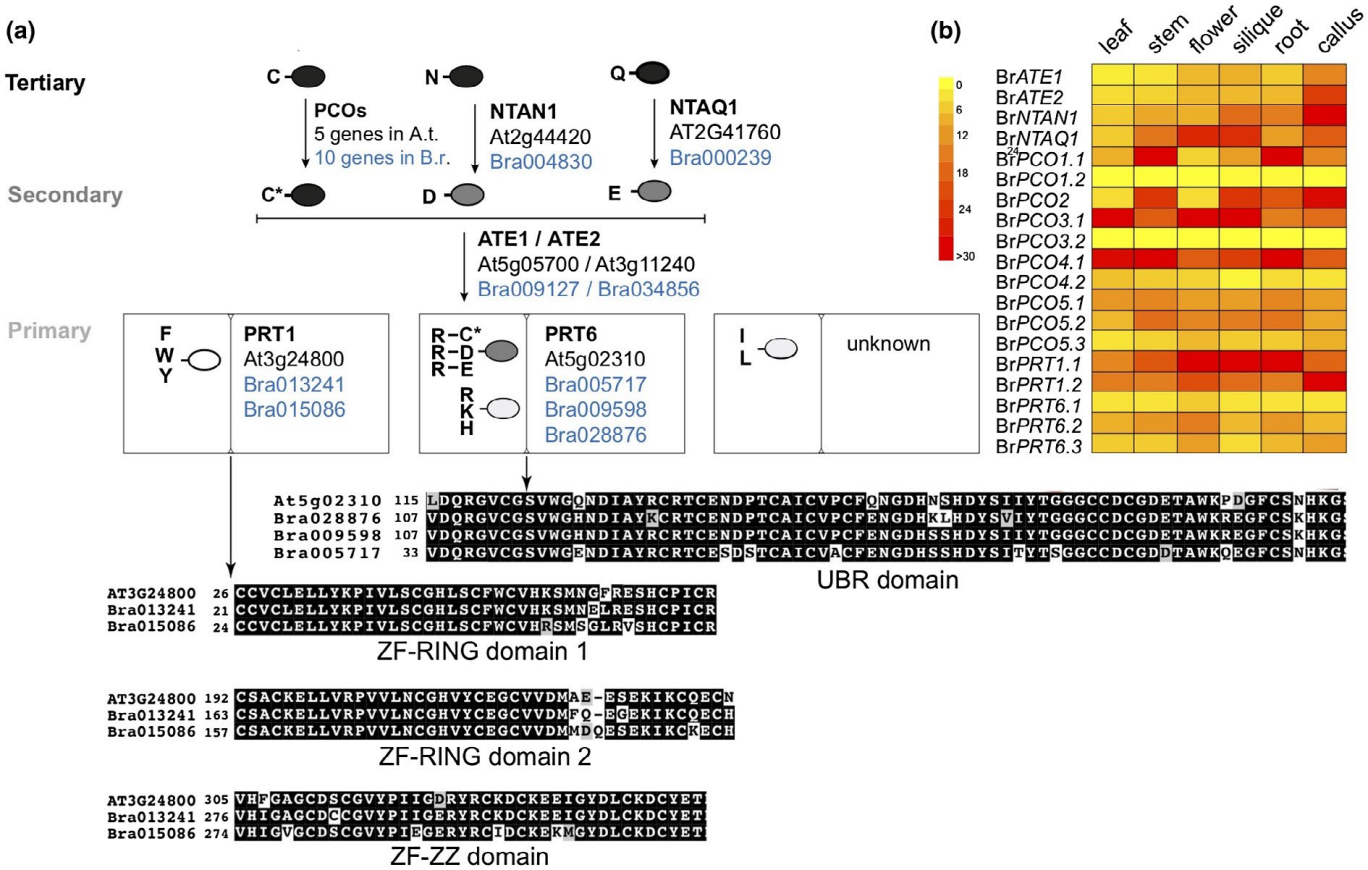
Conservation of N‐degron pathway enzymatic components in *Brassica rapa*. (a) Protein sequences from *Arabidopsis thaliana* N‐degron pathway enzymatic components were used as query in a BLASTp search to identify *B. rapa* homologs. The ovals denote proteins, with single letters indicating the N‐terminal destabilizing residue. The abbreviated names of the enzymatic components are specified, as well as the genome identifier numbers in *A. thaliana* (in black fonts) and in *B. rapa* (blue fonts). C*: oxidized cysteine; PCOs: PLANT CYSTEINE OXIDASEs; NTAN1: N‐terminal Asn amidohydrolase; NTAQ1: N‐terminal Gln amidohydrolase; ATE1/ATE2: Arg‐transferases 1 and 2, respectively; PRT1: PROTEOLYSIS1; PRT6: PROTEOLYSIS6. The identity of the N‐recognin recognizing N‐terminal Leu and Ile remains unknown. The genome identifiers for the 10 *B. rapa PCO* homologs are as follows: *BrPCO1.1* = Bra006280; Br*PCO1.2* = Bra008720; Br*PCO2* = Bra025636; Br*PCO3.1* = Bra025900; Br*PCO3.2* = Bra038171; Br*PCO4.1* = Bra004726; Br*PCO4.2* = Bra000271; Br*PCO5.1* = Bra00741; Br*PCO5.2* = Bra01456; and Br*PCO5.3* = Bra030926. Alignment of the functional domains of PRT1 (ZF‐RING domain 1, ZF‐RING domain 2 and ZF‐ZZ domain) and PRT6 (UBR domain) homologs. The full‐length protein alignments of PRT6 and PRT1 homologs are presented in Figures S4 and S5, respectively. (b) Heatmap showing the expression atlas of N‐degron pathway components in *B. rapa*. The tissue‐specific gene expression of the N‐degron pathway‐related genes in *B. rapa* (accession Chiifu‐401‐42) was obtained from a previously published dataset (GEO43245) (Tong et al., 2013). Scale: fragments per kilobase of exon model per million reads mapped (FPKM) values for each indicated gene

We first verified that the Ub‐X‐LUC reporter was deubiquitylated following transient expression in *B. rapa* by comparing the relative size of the protein after expression in *B. rapa* or in *E. coli*, which lacks deubiquitylating enzymes. Our results indicated that as expected, the Ub‐X‐LUC was deubiquitylated in *B. rapa* (Figure 4b and Figure S2). Next, we chose 10 N‐degron reporter constructs with N‐terminal residues that would be representative of the different types of destabilizing N‐terminal residues found in *Arabidopsis* (Figure 5a), including a basic primary destabilizing residue (Arg; recognized by AtPRT6; Garzon et al., 2007), a hydrophobic primary destabilizing residue (Trp; bound by AtPRT1; Potuschak et al., 1998; Stary et al., 2003), a secondary destabilizing residue (Asp; modified by Arg‐transferases; Graciet et al., 2009; Yoshida et al., 2002), and the different types of tertiary destabilizing residues (Asn and Gln, which are deamidated by AtNTAN1 and AtNTAQ1; Graciet et al., 2010, respectively, as well as Cys, which is oxidized by AtPCO enzymes; Weits et al., 2014, White et al., 2017). A selection of N‐degron reporters bearing presumed N‐terminal stabilizing residues (Met, Ser, Ala, and Thr) were also included.

Three days after infiltration, leaf discs were collected to measure LUC and GUS activities using quantitative enzymatic assays. All LUC activity values were then normalized to the respective GUS activities so that the stability of the X‐LUC reporters could be compared despite any variations in expression levels, which are inherent to transient expression approaches (Figure 4c and Figure S3). As expected based on the identity of N‐terminal destabilizing residues in *Arabidopsis*, we found that Met, Ser, Ala, and Thr were stabilizing residues

, whereas Cys, Gln, Asn, Asp, Arg, and Trp served as N‐terminal destabilizing residues. Using N‐terminal Met and Arg as models, we also verified that the LUC enzymatic activities reflected protein levels using immunoblot analyses with antibodies directed against LUC or GUS (Figure 4d and Figure S2). In sum, our transient expression system is sufficiently sensitive and reproducible to study protein degradation in *B. rapa* using quantitative enzymatic assays, as well as immunoblot analyses, and was applied to identify destabilizing N‐terminal residues in *B. rapa*.

### 3.4 Evolutionary conservation of N‐degron pathway enzymatic components in *B. rapa*

The conservation of N‐terminal destabilizing residues in *B. rapa* suggests that enzymatic components of the N‐degron pathway are also likely to be conserved. We used BLASTp analysis and the protein sequences of all known PRT6 and PRT1/N‐degron enzymatic components in *Arabidopsis* to identify their homologs in *B. rapa*. As expected based on the identity of the destabilizing residues (Figure 4), homologs could be identified for all known enzymatic components (Figure 5a). Interestingly, there appeared to be more diversity in the *B. rapa* genome at the N‐recognin level, in that the *Arabidopsis* genome codes for only one N‐recognin specific for hydrophobic aromatic residues (AtPRT1) and one N‐recognin for basic residues (AtPRT6), while the *B. rapa* genome codes for 2 and 3 homologs of At*PRT1* and At*PRT6*, respectively (Figure 5a). Alignment of known functional domains of these N‐recognins indicates that they are well conserved within Brassicaceae, and are hence likely to execute the same functions in *B. rapa* as in *Arabidopsis* (Figure 5a and Figures S4 and S5). The diversification at the N‐recognin level may instead be relevant in the context of their temporal and/or spatial expression patterns, or in response to specific environmental cues. Therefore, we used publicly available transcriptomics datasets (Tong et al., 2013) to determine the expression atlas of N‐degron pathway‐related genes during development in *B. rapa*. Aside from Br*PCO1.2* and Br*PCO3.2*, which are either expressed at low levels or not expressed, all other N‐degron pathway‐related genes were expressed broadly in different tissues of *B. rapa*, albeit with some differences (Figure 5b). For example, Br*PCO1.1*, an enzymatic component that is important for the oxygen and nitric oxide‐mediated oxidation of N‐terminal cysteine, appears to be more strongly expressed in stem and root compared to leaf, flowers, and siliques.

## 4 DISCUSSION

Here, we show that seedling co‐cultivation with *Agrobacterium* and leaf agroinfiltration allow transient expression of transgenes in *B. rapa*. While seedling co‐cultivation remained inefficient, with only few cells in cotyledons expressing the GUS reporter gene, agroinfiltration of leaves resulted in robust expression throughout the infiltrated area. The primary advantages of this agroinfiltration protocol compared to stable transformation methods of *B. rapa* or *B. napus* (and potentially *B. oleracea* as well) are its ease of use without the need for tissue culture and regeneration, and the rapidity with which results can be obtained. Indeed, 4‐ to 5‐week‐old plants are used, by‐passing the multiple generations that may be necessary to isolate and characterize stable transformants. Developing an efficient and simple transient expression protocol in *Brassica* crops also allows the community to dissect molecular mechanisms in a homologous system, as opposed to relying on heterologous transient expression in *N. benthamiana*, for example, which may not have (i) *Brassica*‐specific proteins or cofactors needed to study the proteins of interest or (ii) metabolic pathways of interest (e.g., glucosinolate biosynthesis pathways, which lead to the production of *Brassica*‐specific secondary metabolites). Notably, the agroinfiltration protocol is also applicable to *B. napus*, which can greatly facilitate potential applications to this essential crop. It should be noted however that the level of transient expression attainable in *B. napus* using this method appears to be lower compared with *B. rapa* based on a qualitative observation of the GUS staining data. In addition to this technical advance, our study indicates that the *Arabidopsis* UBQ3 promoter (Worley et al., 1998) is also suitable for use in *B. rapa*, and presumably in other *Brassica* crops. This adds to the list of constitutive promoters that have been used previously, such as the Cauliflower Mosaic Virus 35S promoter (also used in this study, including an enhanced version of the promoter), the *Arabidopsis* U626 promoter, and a constitutive promoter of the Cassava Vein Mosaic Virus (Lawrenson et al., 2015). The plasmids used in this study may also serve as a resource for future expression studies in *B. rapa* and *B. napus* following removal of the N‐degron reporter and cloning of the construct of interest. Applying this protocol to the determination of N‐terminal destabilizing residues of the N‐degron pathway also showed that it is reproducible and robust, and can be used in conjunction with a range of biochemical and molecular techniques.

The agroinfiltration protocol nevertheless has some limitations. For example, it can only be used to study processes in leaves, which can be a problem to dissect root‐specific pathways. In such cases, protocols such as the *Agrobacterium* root infection procedure adapted by Zhong *et al*. may be useful (Zhong et al., 2016). To test the influence of varying growth conditions on the efficiency of expression, we also applied the protocol to 3‐ to 4‐week‐old *B. rapa* plants grown in greenhouse conditions (16‐hr light/8‐hr dark; 25°C). Our results showed that the proposed protocol also resulted in efficient expression of the GUS reporter in the more variable environmental conditions of a greenhouse, albeit with less reproducibility (data not shown). Furthermore, a comparison of GUS and X‐LUC accumulation after agroinfiltration of *B. rapa* or *N. benthamiana* leaves also indicated that levels of expression are much lower in *B. rapa* (data not shown), which may be problematic for applications that require high levels of protein. Furthermore, as with any transient expression protocol, optimum *Agrobacterium* density and duration of expression need to be specifically determined for the protein of interest. Using GUS as a reporter protein, we could however show that expression can start within 24 hr of agroinfiltration and be maintained for up to 4 days, indicating that transgene expression can be sustained for a considerable period of time.

The agroinfiltration protocol was successfully applied to uncover the sets of destabilizing N‐terminal residues of the N‐degron pathway in *B. rapa*. Notably, the stability conferred by all amino acids tested relative to N‐terminal Met matched the sets of stabilizing and destabilizing amino acids previously uncovered in *Arabidopsis* and tobacco (Graciet et al., 2010). Combined with the identification of the N‐degron pathway enzymatic components in *B. rapa*, these results suggest broad conservation of the N‐degron pathway in *Brassicaceae* and lay the foundations to study the biological functions of this pathway in *Brassica* crop species. Notably, considering that the N‐degron pathway functions as an integrator of environmental signals (Miricescu et al., 2018), this study could facilitate efforts to improve the robustness of *Brassica* crops.

## AUTHORS CONTRIBUTIONS

B.C.M and E.G. designed the work, conducted experiments, and wrote the manuscript.

## Supporting information

Supplementary Material
