# Supplementary material for: A simple and efficient Agrobacterium‐mediated transient expression system to dissect molecular processes in Brassica rapa and Brassica napus
Source: Plant Direct. 2020 Jul 6;4(7):e00237. doi: 10.1002/pld3.237 (PMC7403836; doi:10.1002/pld3.237)
Supplement: Supplementary file 1 — Supplementary Material [file PLD3-4-e00237-s001.docx]

**Supplemental Materials**

**A simple and efficient *Agrobacterium*-mediated transient expression system to dissect molecular processes in *Brassica rapa* and *Brassica napus***

Brian C. Mooney^1^ and Emmanuelle Graciet^1,2*^

^1^ Maynooth University, Department of Biology, Maynooth, Co. Kildare, Ireland

^2^ Kathleen Lonsdale Institute for Human Health Research, Maynooth University, Maynooth, Co. Kildare, Ireland

**Table S1: List of plasmids used in this study**

| Plasmid name | Plasmid number | Reference |
| --- | --- | --- |
| pML-BART UBQ3_pro_:Ub-Gly-LUC 35S_pro_:GUS | pEG378 | (Graciet *et al.*, 2010) |
| pML-BART UBQ3_pro_:Ub-Met-LUC 35S_pro_:GUS | pEG356 | (Graciet *et al.*, 2010) |
| pML-BART UBQ3_pro_:Ub-Ala-LUC 35S_pro_:GUS | pEG385 | (Graciet *et al.*, 2010) |
| pML-BART UBQ3_pro_:Ub-Thr-LUC 35S_pro_:GUS | pEG370 | (Graciet *et al.*, 2010) |
| pML-BART UBQ3_pro_:Ub-Cys-LUC 35S_pro_:GUS | pEG379 | (Graciet *et al.*, 2010) |
| pML-BART UBQ3_pro_:Ub-Gln-LUC 35S_pro_:GUS | pEG374 | (Graciet *et al.*, 2010) |
| pML-BART UBQ3_pro_:Ub-Asn-LUC 35S_pro_:GUS | pEG384 | (Graciet *et al.*, 2010) |
| pML-BART UBQ3_pro_:Ub-Asp-LUC 35S_pro_:GUS | pEG373 | (Graciet *et al.*, 2010) |
| pML-BART UBQ3_pro_:Ub-Arg-LUC 35S_pro_:GUS | pEG368 | (Graciet *et al.*, 2010) |
| pML-BART UBQ3_pro_:Ub-Trp-LUC 35S_pro_:GUS | pEG375 | (Graciet *et al.*, 2010) |
| pCAMBIA2201 |  |  |
| pET22b His_6_ | pEG429 | This study |
| pET22b His_6_-Ub-Met-LUC | pEG432 | This study |

**Table S2: List of oligonucleotides used in this study**

| Oligo# | Sequence (5’ -> 3’) |
| --- | --- |
| His6_up | TATGCACCATCACCATCATCATTC |
| His6_lo | CATGGAATGATGATGGTGATGGTGCA |


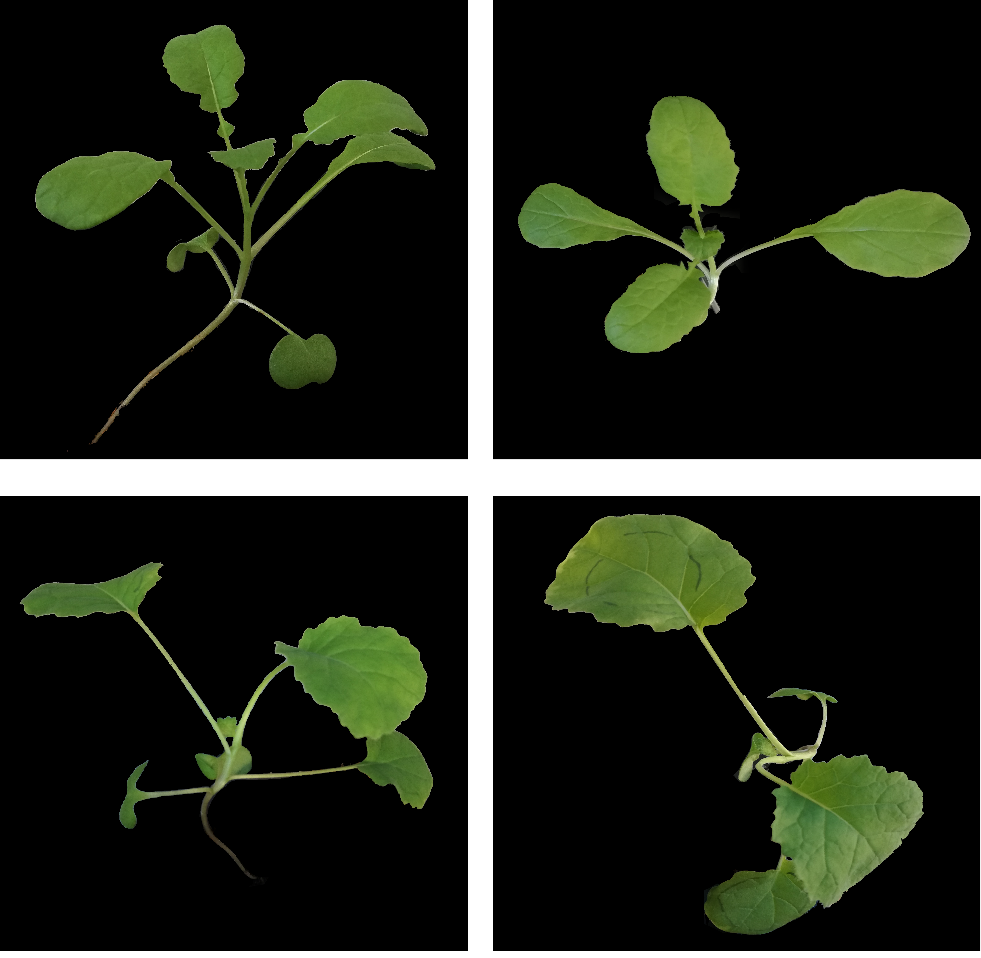


**Supplemental Figure S1: Representative images of the developmental stage of *B. rapa* (upper panels*)* and *B. napus* (lower panels) plants at the time of agroinfiltration.** As indicated in Materials and Methods, seeds were germinated on soil and grown in continuous light conditions (24 h light) at 20°C for 7 days before being transferred to short-day conditions (10 h light/14 h dark) at 22°C for a further 3-4 weeks.


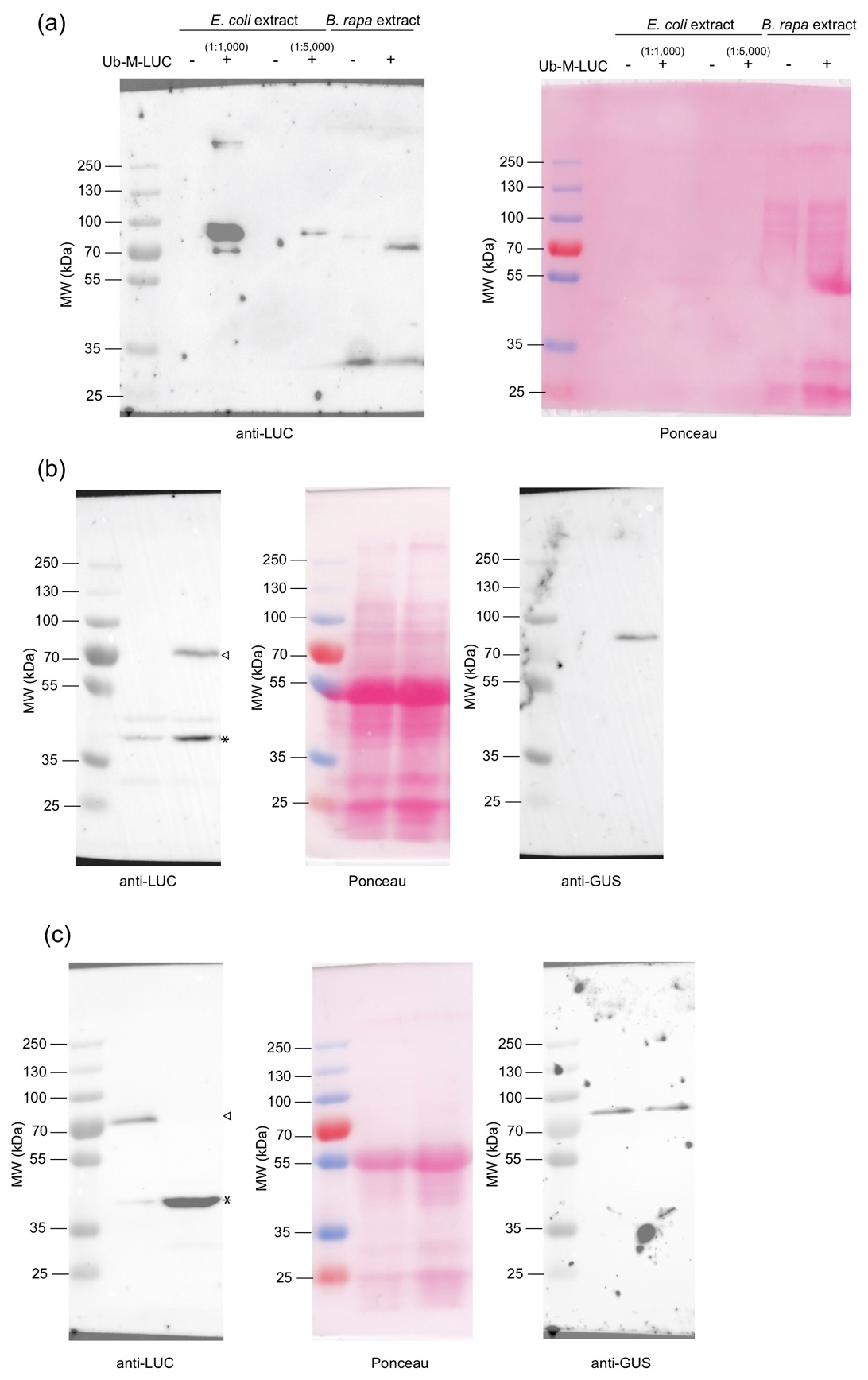


**Supplemental Figure S2: Original blots shown in Figure 4.**

**(a)** *In vivo* deubiquitylation of Ub-Met-LUC (corresponding to immunoblot in Figure 4b). A polyhistidine-tagged His_6_-Ub-Met-LUC was expressed in *E. coli* to serve as a control for a non-deubiquitylated N-degron reporter. Different dilutions of the crude *E. coli* protein extract were loaded (1:1,000 and 1:5,000) to ensure that the signal for the LUC fusion would be comparable to that obtained for expression of Ub-Met-LUC in *B. rapa* and allow a single exposure time. Because of the dilution factor for *E. coli* extract, no proteins are visible on the Ponceau stain of the membrane. Ub-Met-LUC was expressed in *B. rapa* as indicated in Materials and Methods. Tissue collected 3 d after agroinfiltration. Expected molecular weights: His_6_-Ub-Met-LUC: 72 kDa; Ub-Met-LUC: 71 kDa; Met-LUC: 62 kDa. Molecular weight (MW) of the ladder proteins are indicated (PageRuler Plus pre-stained). Results are representative of at least 3 independent replicates.

**(b)** Immunoblot analysis of LUC and GUS protein levels for Met-LUC expression (corresponding to immunoblots in Figure 4d, left panels). Approximately equal protein amounts were loaded to determine potential cross-reacting proteins in *B. rapa* infiltrated with *Agrobacterium* transformed with an empty vector (e.v.) or pEG356 (coding for UBQ3_pro_:Ub-Met-LUC 35S_pro_:GUS). Ponceau staining was performed before immunoblotting with anti-LUC. The membranes were then stripped and incubated with a GUS-specific antibody. The 70-kDa marker protein is fainter after stripping. Note the presence of a cross-reacting protein around 40 kDa, which is detected in empty vector control samples and leaf tissue expressing Ub-X-LUC. Results are representative of at least 3 independent replicates. Open triangle: X-LUC; asterisk: cross-reacting protein.

**(c)** Comparison of Met-LUC and Arg-LUC signal intensities (corresponding to immunoblots in Figure 4d, right panels) for equal GUS levels after infiltration with *Agrobacterium* transformed with pEG356 (UBQ3_pro_:Ub-Met-LUC 35S_pro_:GUS) or pEG368 (UBQ3_pro_:Ub-Arg-LUC 35S_pro_:GUS). Ponceau staining was performed before immunoblotting with anti-LUC. The membranes were then stripped and incubated with a GUS-specific antibody. The 70-kDa marker protein is fainter after stripping. Results are representative of at least 3 independent replicates.


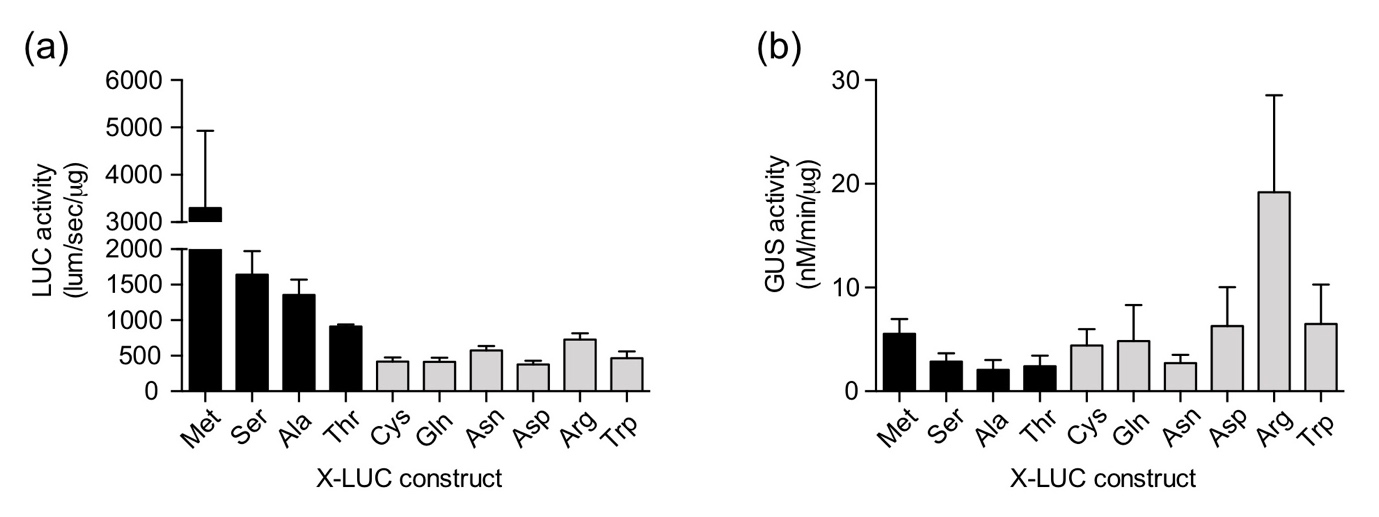


**Supplemental Figure S3: Uncorrected LUC and GUS activities.**

(a) Average non-normalized LUC activities. Error bars represent standard errors of 4 independent replicates, except for Asn, for which 2 independent replicates were conducted. Black and grey bars correspond to stabilizing and destabilizing N-terminal residues, respectively.

(b) Average GUS activities for each of the constructs indicated. Error bars represent standard errors of 4 independent replicates, except for Asn, for which 2 independent replicates were conducted. Black and grey bars correspond to stabilizing and destabilizing N-terminal residues, respectively.


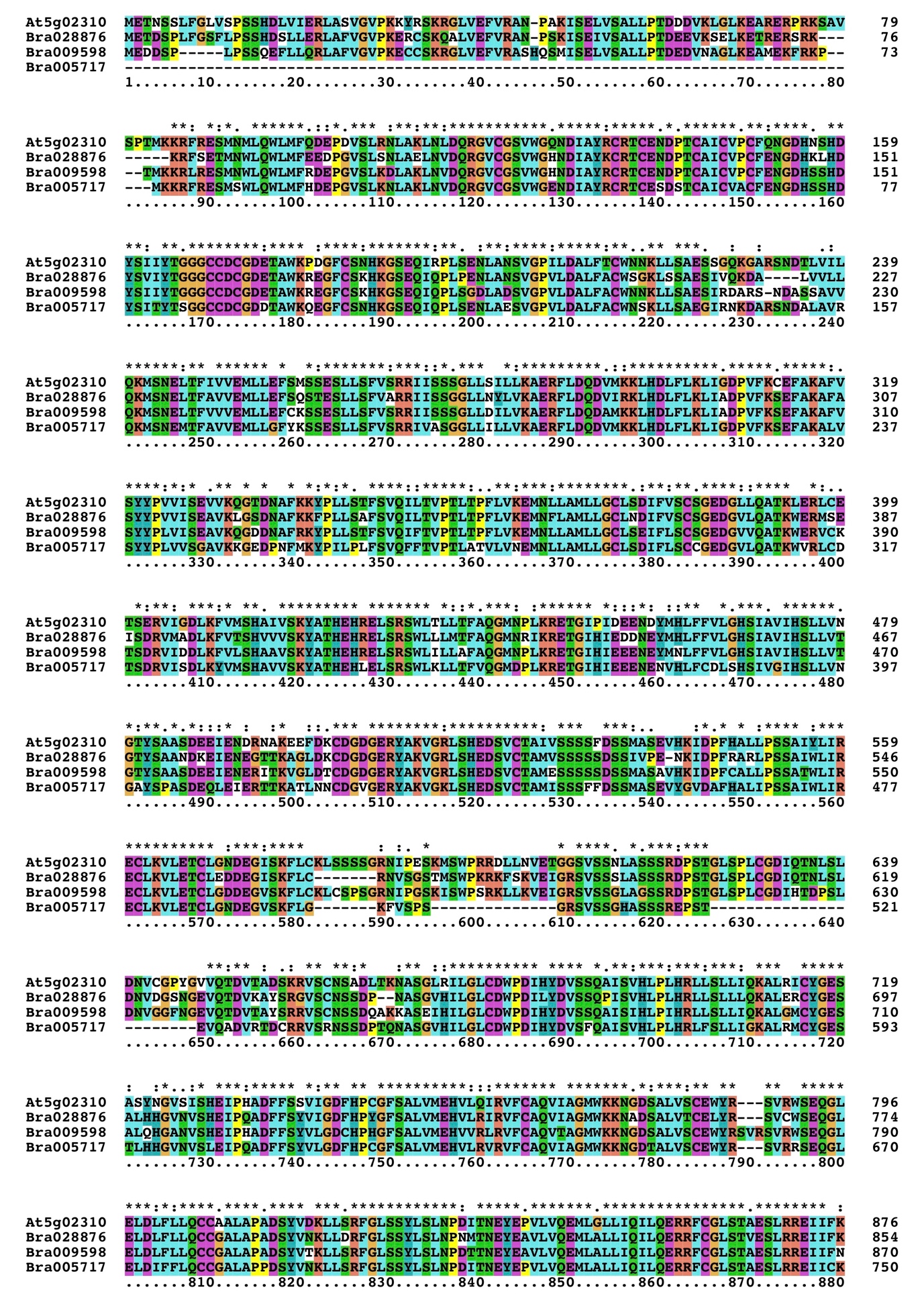


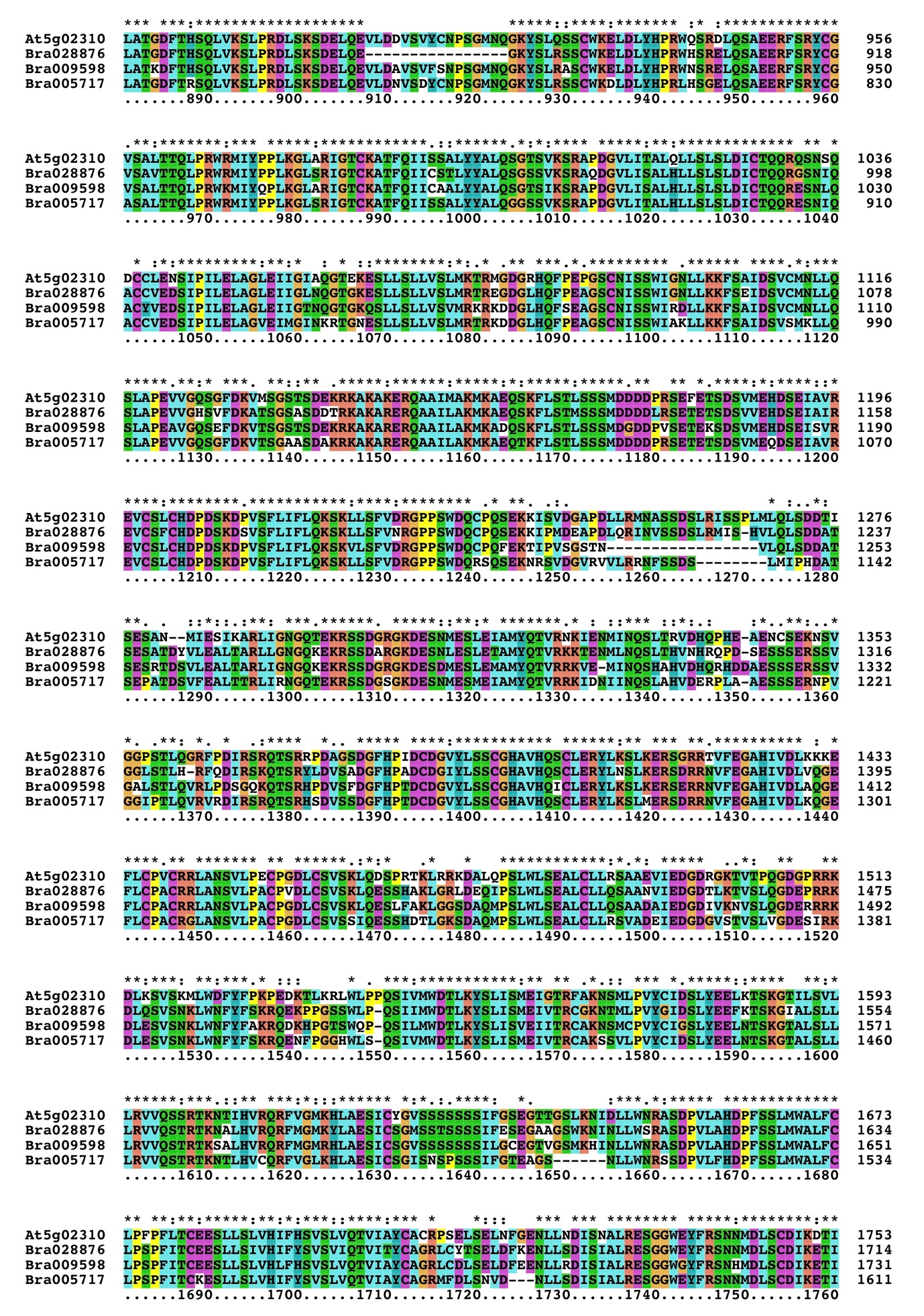


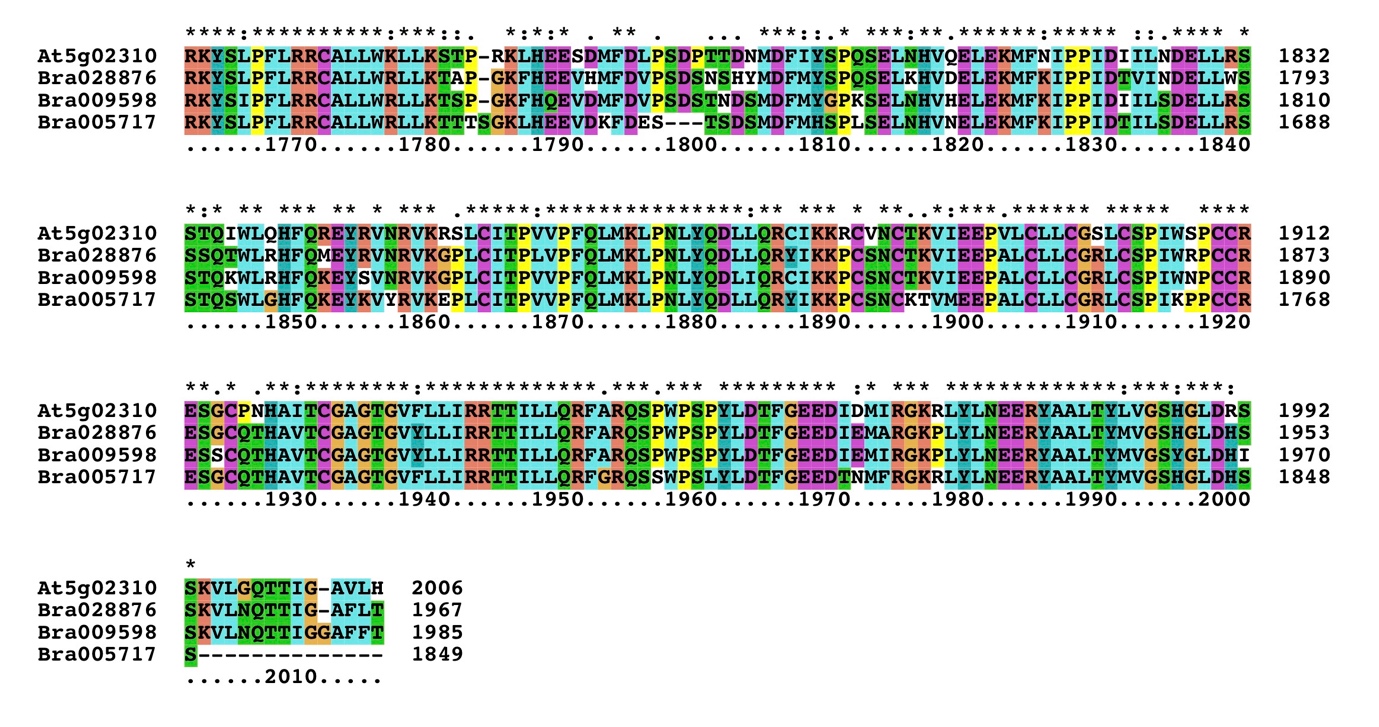


**Supplemental Figure S4: alignment of full-length PRT6 protein sequences from *A. thaliana* and *B. rapa*.**

*A. thaliana* PRT6 (At5g02310) sequence was used as a query in a BLASTp analysis to identify *B. rapa* PRT6 homologs (Bra028876, Bra009598 and Bra005717). Sequences were aligned using ClustalX.


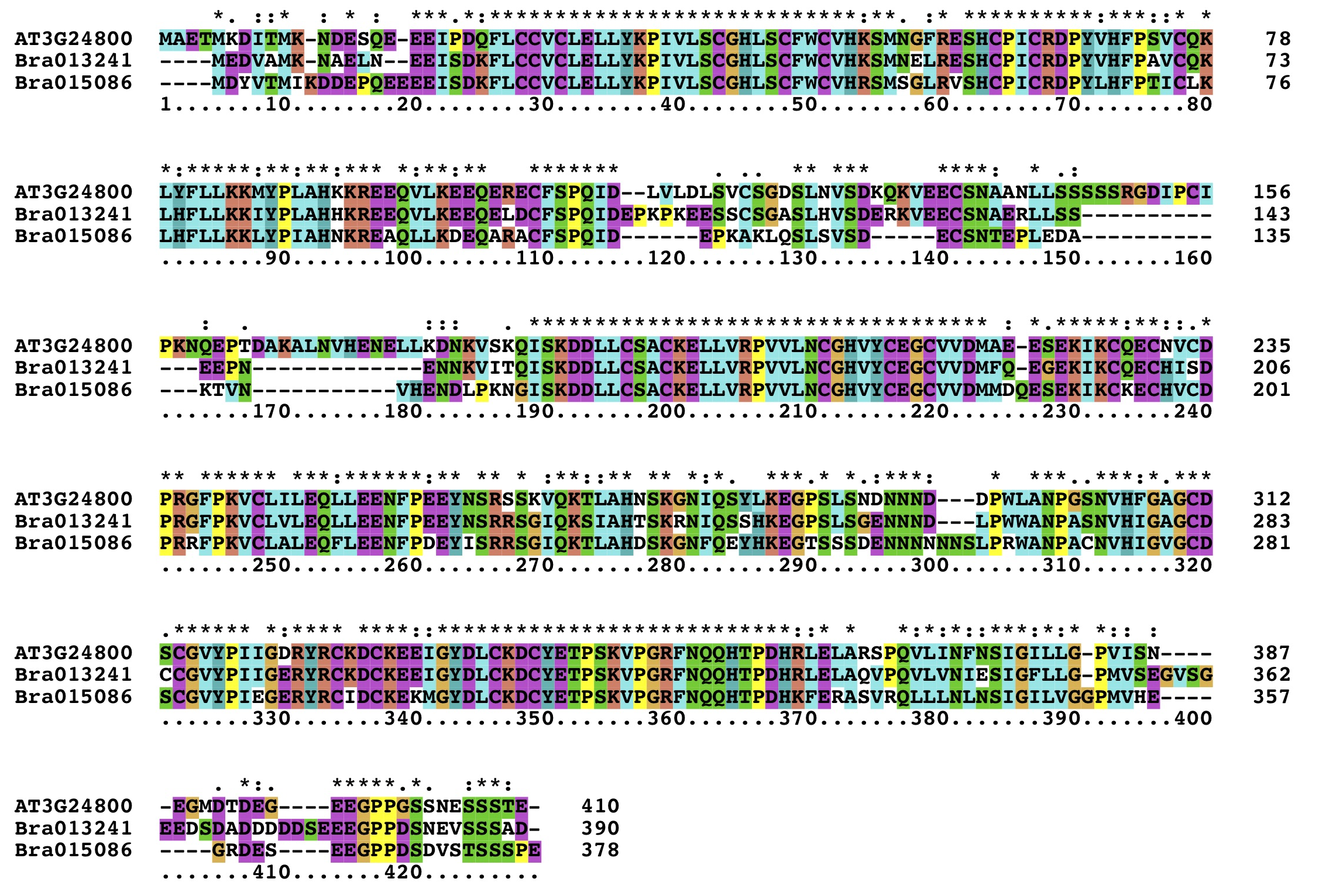


**Supplemental Figure S5: Alignment of full-length PRT1 protein sequences from *A. thaliana* and *B. rapa*.**

*A. thaliana* PRT1 (At3g24800) sequence was used as a query in a BLASTp analysis to identify *B. rapa* PRT1 homologs (Bra013241 and Bra015086). Sequences were aligned using ClustalX.

**References**

**Graciet, E., Mesiti, F. and Wellmer, F.** (2010) Structure and evolutionary conservation of the plant N-end rule pathway. *Plant J.*, **61**, 741-751.
